# Long-term oyster shell powder applications increase crop yields and control soil acidity and cadmium in red soil drylands

**DOI:** 10.3389/fpls.2025.1506733

**Published:** 2025-02-28

**Authors:** Hao Li, Yan Wu, Jiwen Li, Tianfu Han, Kailou Liu, Shangshu Huang

**Affiliations:** ^1^ Jiangxi Institute of Red Soil and Germplasm Resources, Jiangxi Province Key Laboratory of Arable Land Improvement and Quality Enhancement, Nanchang, Jiangxi, China; ^2^ Institute of Agricultural Resources and Regional Planning, Chinese Academy of Agricultural Sciences, Beijing, China; ^3^ School of Agricultural Sciences, Zhengzhou University, Zhengzhou, China

**Keywords:** oyster shell powder, red soil drylands, soil acidification, cadmium, peanut

## Abstract

The intensification of agricultural production has significantly reduced land availability, necessitating continuous cropping cycles that degrade soil quality and inhibit crop growth. While the short-term use of soil amendments has shown significant potential for mitigating these challenges, few studies have explored their long-term effects on acidified soils and heavy metal accumulation. Between 2013 and 2018, a field experiment was conducted in the peanut (*Arachis hypogaea L*)-growing region of Jinxian County, Jiangxi Province, to investigate the long-term effects of oyster shell powder applied to upland red soil. Before the experiment, the soil properties were as follows: pH, 4.54, total soil cadmium (Cd) content, 0.49 mg kg^-^¹; and available Cd content, 0.25 mg kg^-^¹. The experiment included three treatments combining chemical fertilizers with oyster shell powder at application rates of 750, 1500, and 2250 kg ha^-^¹ (L750, L1500, L2250) and a control with only chemical fertilizer (L0). From 2013 to 2018, peanut yield among all treatments was assessed at maturity. Soil pH was then measured using a pH meter with a 2.5:1 water-to-soil ratio. Exchangeable hydrogen and aluminum were determined using the potassium chloride exchange-neutralization titration method. Meanwhile, available Cd content was extracted using 0.1 M CaCl_2_ and measured with a flame atomic absorption spectrophotometer. While all treatments showed an annual decline in peanut yield from 2013 to 2018, but oyster shell applications significantly reduced the rate of crop yield decline. Compared to L0, the yields of L750, L1500, and L2250 treatments increased by 5.55%-19.42%, 8.64%-28.74%, and 15.43%-37.01%, respectively. Soil pH values in the L750, L1500, and L2250 treatments were higher than the L0 treatment by 0.03-0.31, 0.16-0.48, and 0.28-0.65 units, respectively. Their exchangeable hydrogen contents decreased by 10.17%-24.24%, 16.67%-27.94%, and 23.40%-29.44%. In addition, exchangeable aluminum contents decreased by 5.05%-26.09%, 23.23%-46.27%, and 39.73%-66.97%. In contrast, soil available Cd contents in the L750, L1500, and L2250 treatments were lower than the L0 treatment by 7.96%-19.29%, 9.56%-30.71%, and 13.94%-34.65%, respectively. Correlation analysis revealed that soil pH was positively associated with peanut yield and negatively correlated with exchangeable hydrogen, exchangeable aluminum, and available Cd. For every 0.1 unit increase in soil pH, peanut yields increased by 119.62-389.82 kg ha^-^¹, while available Cd decreased by 0.06-0.12 mg kg^-^¹. Therefore, these findings demonstrate the efficacy of continuous oyster shell powder application in controlling soil acidification and reducing Cd levels in upland red soil.

## 1 Introduction

Red soil is a key agricultural resource in China, accounting for approximately 28% of the country’s total land area and 11% of the hilly regions of southern China (Xu et al., 2022). Despite its critical role in agriculture, red soils exhibits poor nutrient retention and a naturally low pH, typically lower than 6.5. Over the past 30 years, red soil drylands have experienced widespread degradation and increased acidification, severely affecting sustainable agricultural development (Guo et al., 2010). Soil acidification is directly reflected by a decline in pH, with most acidic soils having a pH value below 5.5. The cultivated soil in Jiangxi Province of China is mostly acidic, and the strongly acidic soil with pH ≤5.5 accounts for 81.05%. As soil acidifies, base ions become scarce and decrease the soil’s buffering capacity (Xie et al., 2023; Li et al., 2024), resulting in environmental issues such as increased heavy metal activation that further poisons the soil. At present, Jiangxi Province of China is facing a severe situation of farmland soil heavy metal pollution prevention and control, the average background cadmium (Cd) of soil is 0.21 mg kg^-^¹, which poses a serious threat to the local ecological environment, agricultural product safety and human health. Therefore, it is necessary to use effective patterns for addressing these problems and challenges.

Several methods have been developed to mitigate soil acidification, such as rational fertilization and the addition of alkaline substances. Numerous studies have shown that applying lime can raise soil pH, neutralizing acidification and leading to increased crop yields. However, excessive lime can cause soil compaction, reduce microbial activity, disrupt the nutrient supply balance, and negatively impact crop yields (Lombi et al., 2003; Barbieri et al., 2015; Eduardo et al., 2018). Recent efforts have focused on developing soil conditioners as a safe, convenient, and effective method of reducing soil acidification (Xu et al., 2006; Bhardwaj et al., 2010). Previous studies have demonstrated that calcium carbonate-based soil conditioners effectively improve soil pH and increase crop yields (Li et al., 2024). Similarly, Zhang He et al. showed that soil amendments derived from seafood waste such as shrimp heads and crab shells improve the soil carbon-to-nitrogen ratio, leading to enhanced crop yields (Zhang et al., 2021). While these studies demonstrate the effects of soil conditioners over 1 to 3 years (Eduardo et al., 2018; Li et al., 2024), research on their long-term effects remains limited.

Oyster shell-based soil amendments offer a promising solution for improving soil characteristics due to their high calcium carbonate content (approximately 95% of total weight). Oysters, a major aquatic product in China, are primarily distributed in coastal regions such as Fujian and Shandong provinces, where discarded shells account for 70% of the mollusks’ total mass, contributing to environmental pollution. Short-term studies have shown that applying oyster shell powder in acidic soil improves pH and increases crop yield due to the calcium boost. However, assessing the long-term impact of these soil conditioners is essential for determining their practical effects on soil ecosystems, crop growth cycles, and soil quality. This study utilizes the calcium-loving crop peanuts to investigate the effects of oyster shell-based soil conditioners in a typical red soil region (Jiangxi Province) over five years. By controlling soil acidification, these treatments are hypothesized to restore soil productivity, ultimately increasing peanut yields. Our results have significant implications for sustainable agricultural development in the region, ensuring regional food security, recycling seafood by-products, increasing farmers’ income, and restoring the degraded ecological environment.

## 2 Materials and methods

### 2.1 Experimental site

The experiment was conducted in Jinxian County, Jiangxi Province (116°17’60’’E, 28°35’24’’N), on red dryland soil derived from Quaternary red clay. The experimental soil is classified as red soil in the Chinese soil classification or as a typical Plinthosol in soil taxonomy according World Reference Base for Soil Resources from FAO, with weak water-holding capacity and medium to low fertility. The region has an average annual temperature of 18.1°C, an accumulated temperature ≥10°C of 6,480°C, and an annual rainfall of 1,537 mm. Before the experiment, the soil properties were as follows: pH, 4.54; exchangeable hydrogen, 4.61 mmol kg^-^¹; exchangeable aluminum, 39.14 mmol kg^-^¹; organic matter, 21.13 g kg^-^¹; total nitrogen, 1.21 g kg^-^¹; total phosphorus, 0.77 g kg^-^¹; total potassium, 8.99 g kg^-^¹; alkaline hydrolyzable nitrogen, 57.50 mg kg^-^¹; available phosphorus, 26.75 mg kg^-^¹; available potassium, 245.05 mg kg^-^¹; total soil Cd content, 0.49 mg kg^-^¹; and available Cd content, 0.25 mg kg^-^¹.

### 2.2 Soil amendment

The soil amendment used in this experiment was oyster shell powder, primarily derived from selected marine oysters and other shells. It was processed through a protective roasting method. The amendment was obtained from Mata (Fujian) Ecological Technology Co., Ltd and features a micro-porous structure of 2-10 μm. The roasting temperature is usually between 950°C and 1150°C, and the roasting time is generally 20 minutes to 50 minutes, so that the organic matter in the oyster shell is decomposed, and the oyster shell after roasting is crushed to obtain the oyster shell powder. The oyster shell powder had a CaO content ≥ 45% and a pH range of 8.5-10.5.

### 2.3 Experimental design

The experiment was conducted over five consecutive years (2013-2018) with four treatments: a chemical fertilizer control with 1050 kg ha^-1^ compound fertilizer (N, P_2_O_5_, and K_2_O at 15%, 15% and 15%) (L0), and a chemical fertilizer combined with oyster shell powder at rates of 750 kg ha^-^¹ (L750), 1500 kg ha^-^¹ (L1500), and 2250 kg ha^-^¹ (L2250). Each treatment was repeated three times, for a total of 12 plots. Plots measured 50 m² (5 × 10 m) and were arranged in a randomized block design.

The peanut variety selected for the experiment was ‘Yueyou 256,’ sown at a rate of 300 kg ha^-^¹, with a plant spacing of 10 × 40 cm and two seeds per hole. Each year, peanuts were planted in early May and harvested in late September. Chemical fertilizers were uniformly applied across all treatments: 70% as a base application and 30% after peanut emergence. The oyster shell powder was applied entirely as basal fertilizers.

### 2.4 Measurement indicators

Peanut yield was assessed at maturity in late September each year, with plots individually harvested and threshed. The harvested peanuts were dried and weighed to calculate yield. After harvesting, soil samples were collected from each plot to determine acidification. Soil pH was measured using a pH meter with a 2.5:1 water-to-soil ratio. Exchangeable hydrogen and aluminum were determined using the potassium chloride exchange-neutralization titration method (Bao, 2022).

Soil samples were further evaluated for available Cd content. Cd was extracted using 0.1 M CaCl_2_ and its contents were measured with a flame atomic absorption spectrophotometer (Ardestani et al., 2013).

For initial soil, the soil pH, exchangeable hydrogen and aluminum, available Cd contents were measured by above method (Ardestani et al., 2013; Bao, 2022). Moreover, soil organic matter was determined in triplicate using the wet oxidation method (Bao, 2022); total nitrogen, total phosphorus, total potassium, alkaline hydrolyzable nitrogen, available phosphorus, available potassium, contents were determined by the method described by book (Bao, 2022). Soil total Cd content was extracted by nitric acid, sulfuric acid and perchloric acid and measured according the method of available Cd (Ardestani et al., 2013). The methods about CaO and pH in oyster shell powder were also described by book (Bao, 2022).

### 2.5 Statistical analysis

All data were maintained using Microsoft Excel 2003. SPSS 16.0 was employed for variance analysis to determine the effects of soil pH, exchangeable hydrogen, and exchangeable aluminum on peanut yield and available Cd content. One-way analysis of variance (ANOVA) was performed to test the significant differences in all treatment for the same year. Tbales and figures were generated using Microsoft Excel and Origin 8.1.

## 3 Results

### 3.1 Effect of oyster shell powder on peanut yield

**Figure 1:**
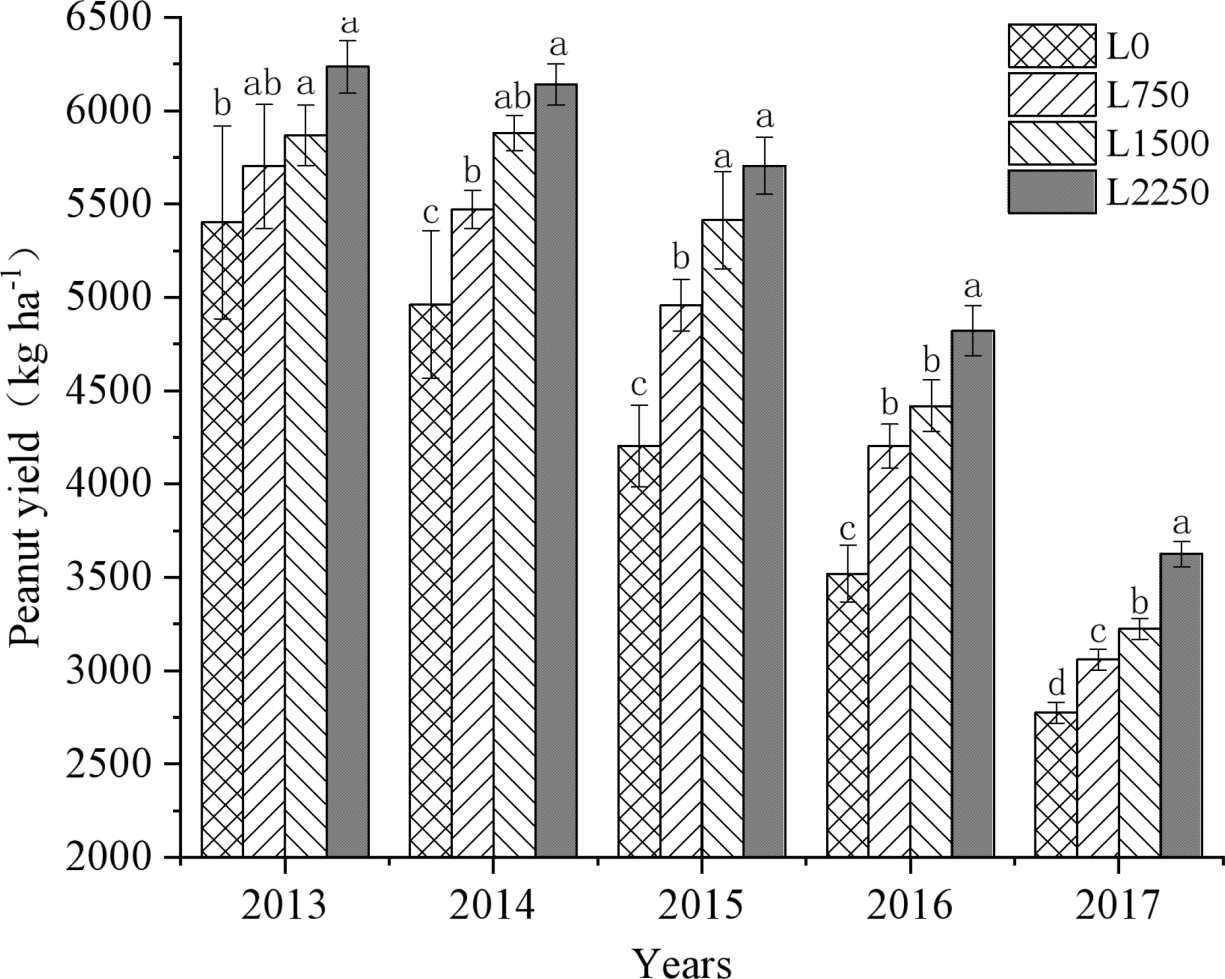
Changes in peanut yield with continuous oyster shell powder application.

Although peanut yields in all treatments decreased annually from 2013 to 2018 due to continuous cropping, the application of oyster shell powder significantly slowed the rate of decline (**Figure 1**). Compared to L0, peanut yields in L750, L1500, and L2250 increased by 5.55%-19.42%, 8.64%-28.74%, and 15.43%-37.01%, respectively, over the five-year period. This yield increase became more pronounced with higher dosages of oyster shell powder. The slope of the fitted equation (**Table 1**) indicates that the continuous application of oyster shell powder was significantly positively correlated with peanut yield (*p* < 0.05). In the first year, each 1 kg ha^-1^ increase in oyster shell powder raised peanut yields by 0.39 kg ha^-1^; however, after five years of continuous application, the yield increase rate slowed to 0.35 kg ha^-1^.

**Table 1:** Fitted equations between oyster shell powder rate and peanut yield.

Year	Equation	R^2^	P
2013	y=5339.02 + 0.39x	0.9654	0.0174
2014	y=5132.45 + 0.47x	0.9767	0.0117
2015	y=4396.57 + 0.61x	0.9528	0.0239
2016	y=3661.16 + 0.53x	0.9421	0.0294
2017	y=2764.97 + 0.35x	0.9706	0.0148

Different lowercase letters indicate significant differences between treatments within the same year (P<0.05).

### 3.2 Effect of oyster shell powder on acidification control

**Figure 2:**
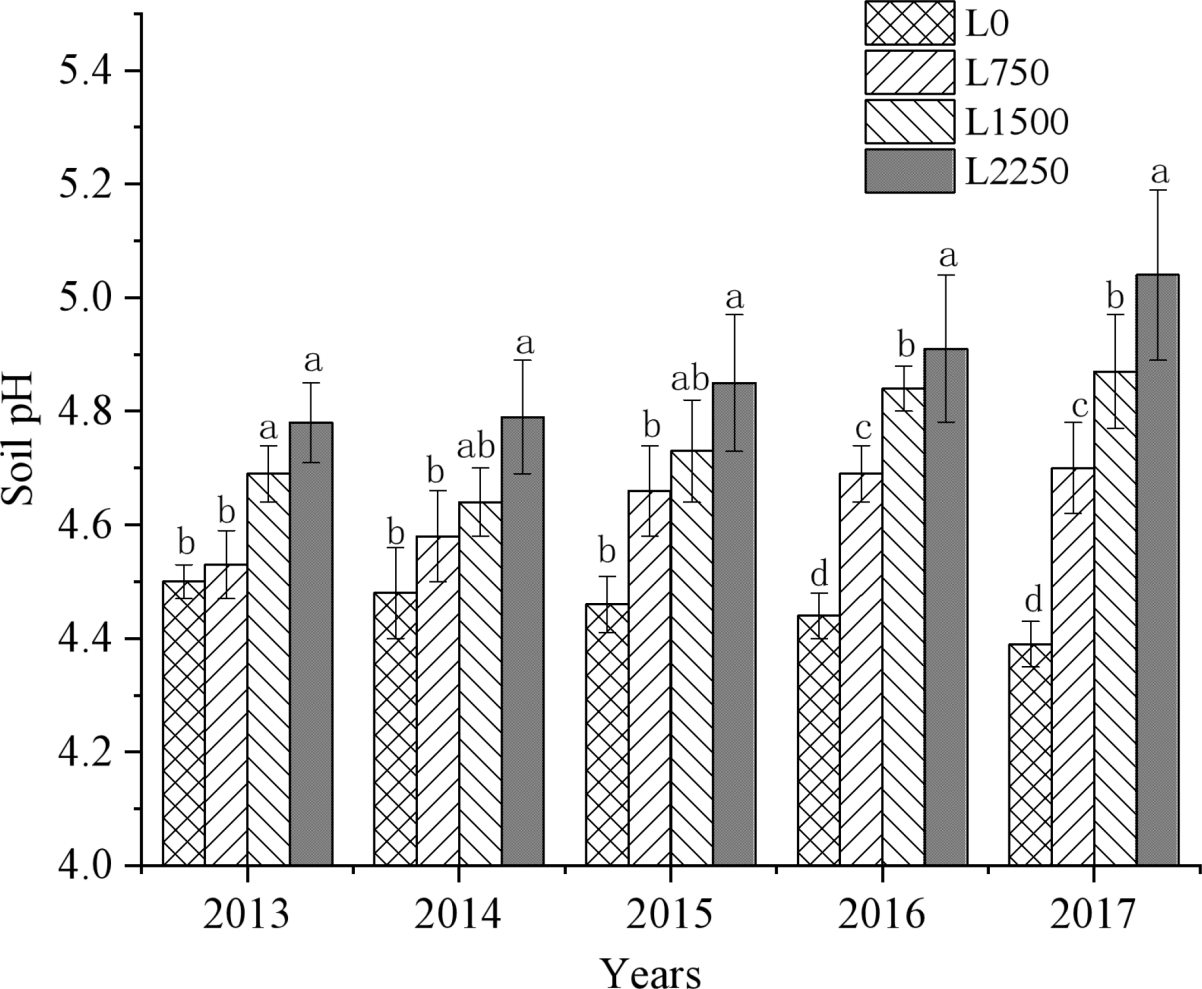
Changes in soil pH with continuous oyster shell powder application.

**Figure 3:**
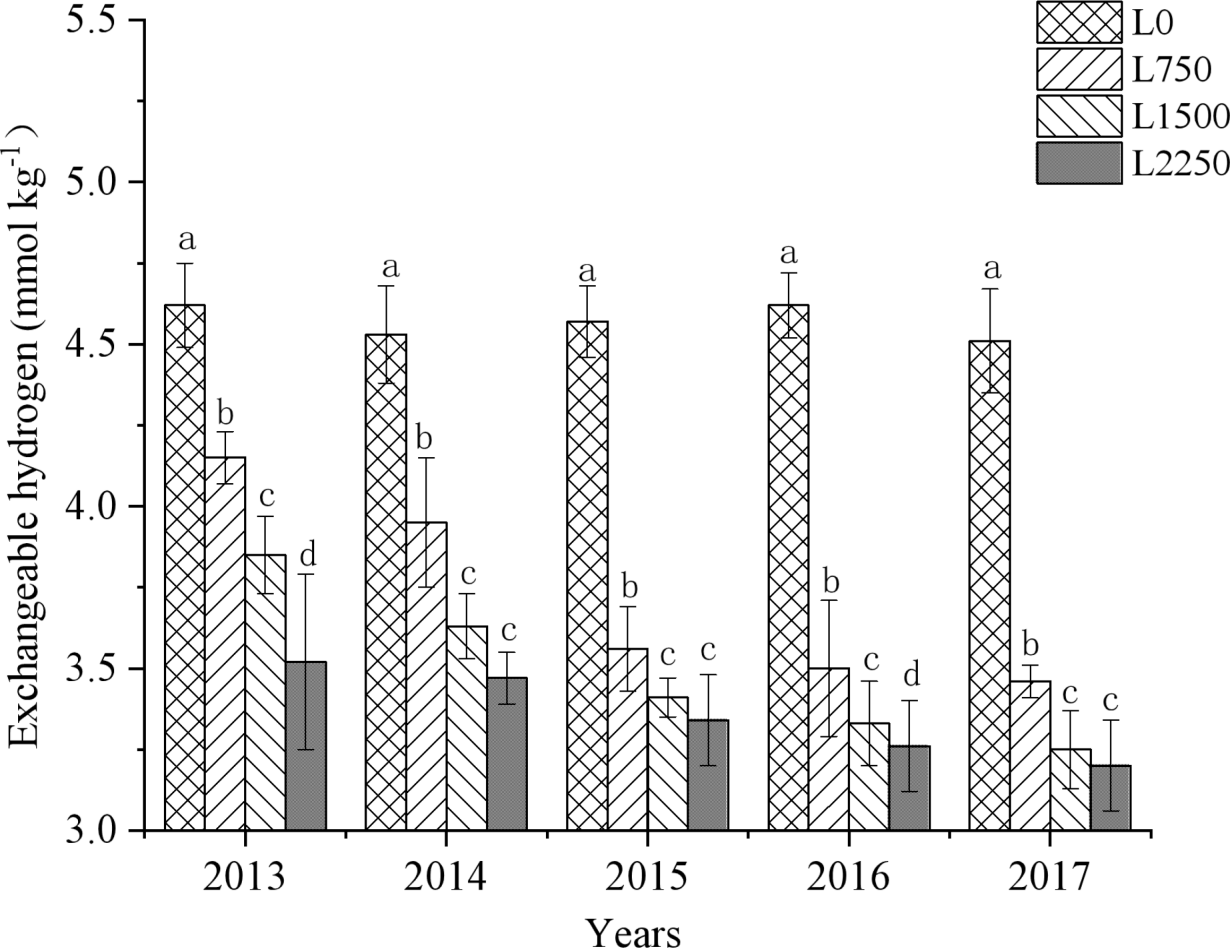
Changes in exchangeable hydrogen with continuous oyster shell powder application.

**Figure 4:**
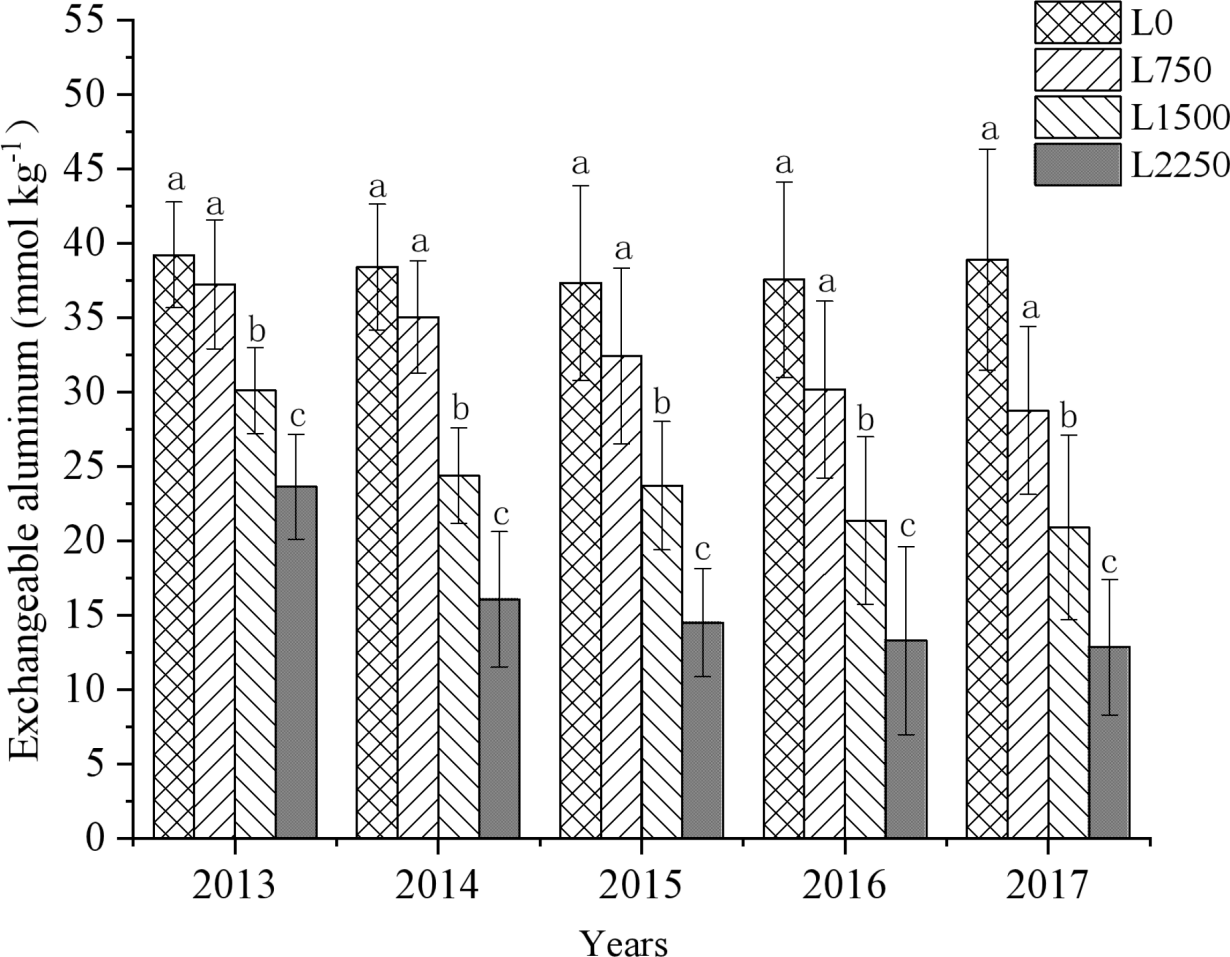
Changes in soil exchangeable aluminum with continuous oyster shell powder application.

Our results show that the application of oyster shell powder in red soil drylands significantly reduced soil acidification (**Figures 2**–**4**). Compared to L0, the soil pH in L750, L1500, and L2250 increased by 0.03-0.31, 0.16-0.48, and 0.28-0.65 units, respectively, over the five years of treatment. Additionally, exchangeable hydrogen decreased by 10.17%-24.24%, 16.67%-27.94%, and 23.40%-29.44%, and exchangeable aluminum decreased by 5.05%-26.09%, 23.23%-46.27%, and 39.73%-66.97%, respectively. The reduction in soil acidification became more pronounced with each year of treatment. The slope of the fitted equations (**Tables 2**–**4**) shows that continuous application of oyster shell powder was significantly positively correlated with soil pH, and negatively correlated with exchangeable hydrogen and aluminum content (*p* < 0.05). After five years of continuous peanut cropping, the average soil pH decreased from 4.49 to 4.40 in L0 treatment. In the first year, each 1,000 kg ha^-1^ increase in oyster shell powder raised soil pH by 0.1, reduced exchangeable hydrogen content by 0.5 mmol kg^-1^, and reduced exchangeable aluminum content by 10 mmol kg^-1^; after five years, the same increase in oyster shell powder raised pH by 0.3, reduced exchangeable hydrogen content by 0.4 mmol kg^-1^, and reduced exchangeable aluminum content by 10 mmol kg^-1^.

**Table 2:** Fitted equations between oyster shell powder rate and soil pH.

Year	Equation	R^2^	P
2013	y=4.49 + 0.0001x	0.9527	0.0242
2014	y=4.47 + 0.0001x	0.9557	0.0224
2015	y=4.47 + 0.0002x	0.9613	0.0195
2016	y=4.46 + 0.0003x	0.9682	0.0160
2017	y=4.40 + 0.0003x	0.9658	0.0114

Different lowercase letters indicate significant differences between treatments within the same year (p<0.05).

**Table 3:** Fitted equations between oyster shell powder rate and soil exchangeable hydrogen.

Year	Equation	R^2^	P
2013	y=4.56-0.0005x	0.9761	0.0120
2014	y=4.40-0.0004x	0.9297	0.0358
2015	y=4.35-0.0006x	0.8119	0.0989
2016	y=4.50-0.0007x	0.8726	0.0659
2017	y=3.86-0.0004x	0.5244	0.2758

Different lowercase letters indicate significant differences between treatments within the same year (p<0.05).

**Table 4:** Fitted equations between oyster shell powder rate and soil exchangeable aluminum.

Year	Equation	R^2^	P
2013	y=40.37-0.01x	0.9634	0.0185
2014	y=40.33-0.01x	0.9606	0.0199
2015	y=39.02-0.01x	0.9869	0.0066
2016	y=37.88-0.01x	0.9988	5.9294
2017	y=37.95-0.01x	0.9965	0.0018

Different lowercase letters indicate significant differences between treatments within the same year (P<0.05).

### 3.3 Effect of oyster shell powder on heavy metal immobilization

**Figure 5:**
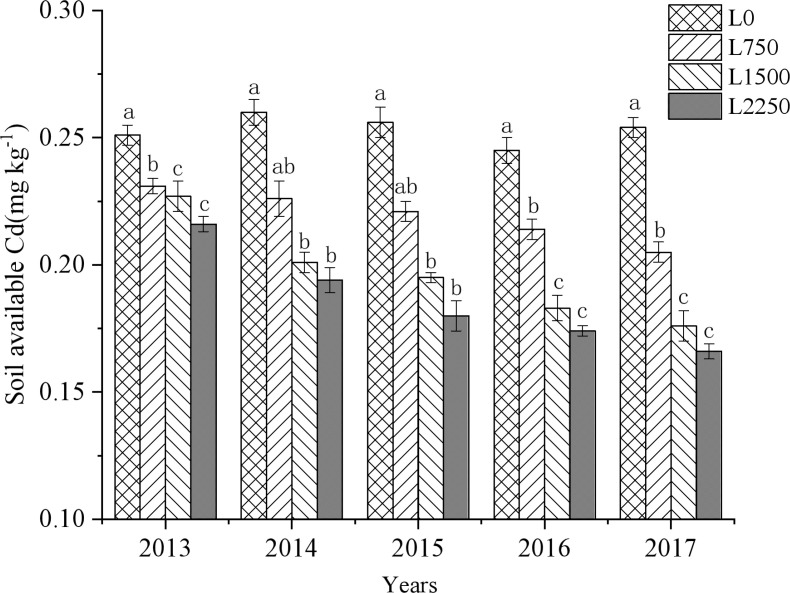
Changes in soil available Cd with continuous oyster shell powder application.

Oyster shell powder applications in red soil drylands significantly reduced available Cd content in the soil. Compared to L0, the available Cd in L750, L1500, and L2250 treatments decreased by 7.96%-19.29%, 9.56%-30.71%, and 13.94%-34.65%, respectively, over the five years (**Figure 5**). These reductions became more pronounced with increasing amounts of oyster shell powder and were further accentuated by application duration. The slope of the fitted equation indicates a negative correlation between continuous treatment and available soil Cd content (*p* < 0.05) (**Table 5**). Across all five years, every 1,000 kg ha^-1^ increase in oyster shell powder reduced available Cd content by 0.01 mg kg^-1^.

**Table 5:** Fitted equations between oyster shell powder rate and soil available Cd.

Year	Equation	R^2^	P
2013	y=0.25-0.00001x	0.9160	0.0429
2014	y=0.25-0.00003x	0.9270	0.0372
2015	y=0.25-0.00004x	0.9631	0.0186
2016	y=0.24-0.00003x	0.9643	0.0180
2017	y=0.24-0.00004x	0.9342	0.0335

Different lowercase letters indicate significant differences between treatments within the same year (p<0.05).

### 3.4 Correlation analysis of soil pH, exchangeable hydrogen, and aluminum with peanut yield and available Cd

**Figure 6:**
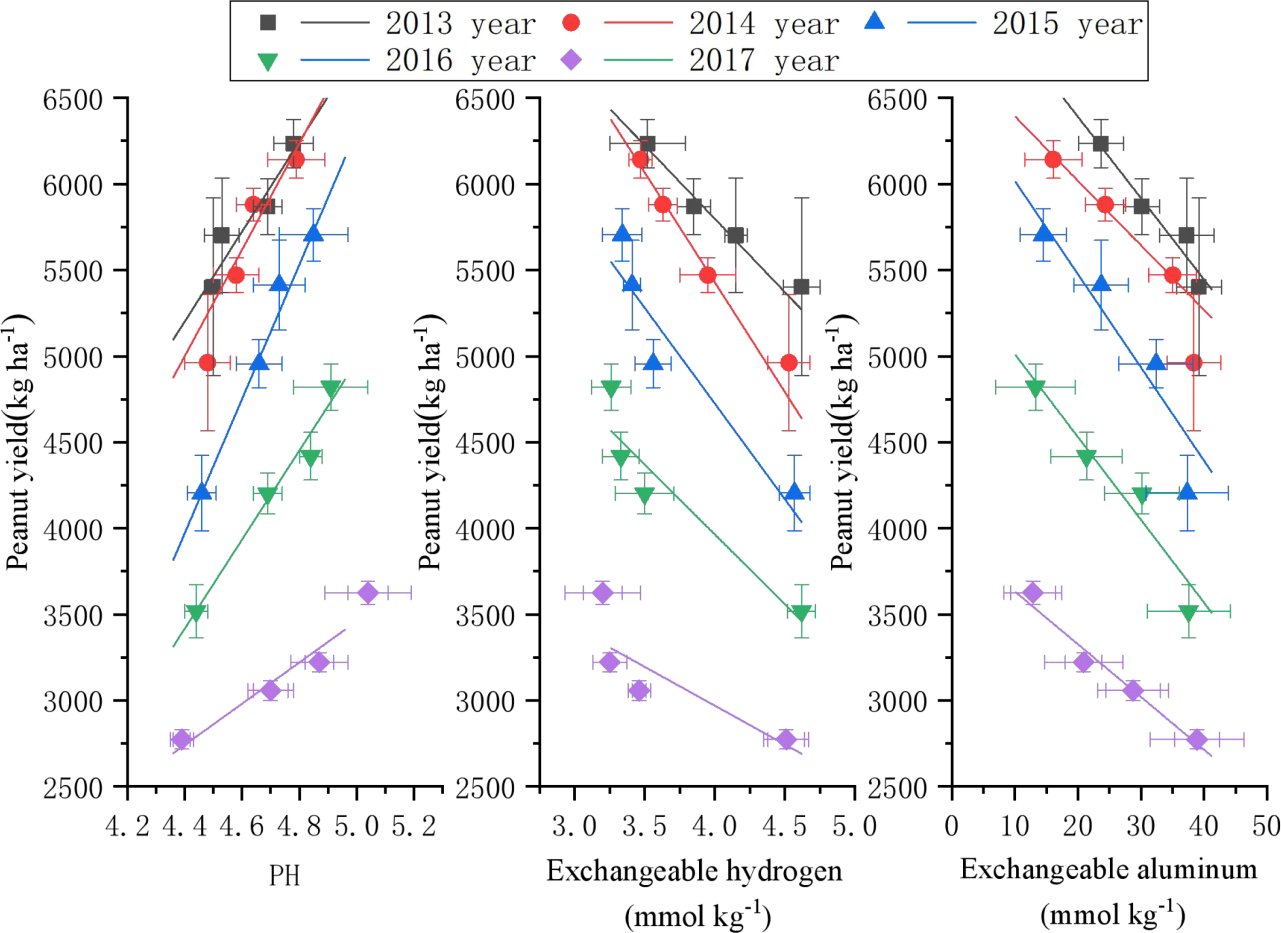
Correlation between peanut yield and soil pH, exchangeable hydrogen, and exchangeable aluminum.

Our soil analysis revealed that peanut yield was positively correlated with soil pH, while it was negatively correlated with exchangeable hydrogen and aluminum (**Figure 6**). Continuous cropping led to an annual decrease in soil pH throughout our experiment. From 2015 to 2017 (**Table 6**), soil pH showed a significant positive correlation with peanut yield (*R^2^* = 0.98, 0.96, 0.94, *p* < 0.05). In contrast, exchangeable hydrogen exhibited a significant negative correlation with peanut yield between 2013 and 2014 (*R^2^* = 0.95, 0.98, *p* < 0.05), although this correlation was not significant in later years (2015 to 2017). Additionally, exchangeable aluminum was significantly correlated with peanut yield in all years (*p* < 0.05), except for 2015 (*p* > 0.05). The slope of the linear equation indicates that for every 0.1 unit increase in pH, peanut yields increased by between 119.62 and 389.82 kg ha^-1^. Additionally, for every 0.1 mmol kg^-1^ increase in exchangeable hydrogen, yields increased by 11.10 to 127.99 kg ha^-1^, while each 0.01 mmol kg^-1^ increase in exchangeable aluminum resulted in yield increases of 3.06 to 5.41 kg ha^-1^.

**Table 6:** Fitted equations between peanut yield and soil pH, exchangeable hydrogen, and exchangeable aluminum.

Indexes	Year	Equation	R^2^	P
pH	2013	y=-6287.19 + 2610.95x	0.8847	0.0594
	2014	y=-8594.90 + 3089.67x	0.8550	0.0754
	2015	y=-13181.26 + 3898.21x	0.9879	0.0061
	2016	y=-7958.19 + 2588.32x	0.9656	0.0174
	2017	y=-2521.32 + 1196.20x	0.9355	0.0328
Exchangeable hydrogen	2013	y=9205.93-851.84x	0.9572	0.0216
	2014	y=10524.97-1273.85x	0.9810	0.0096
	2015	y=9165.05-110.99x	0.8182	0.0955
	2016	y=7205.81-810.17x	0.8529	0.0765
	2017	y=-4772.60-450.59x	0.7045	0.1606
Exchangeable aluminum	2013	y=7335.70-47.25x	0.9540	0.0233
	2014	y=6558.37-54.11x	0.9606	0.0199
	2015	y=6767.91-37.46x	0.8849	0.0593
	2016	y=5491.67-48.06x	0.9244	0.0386
	2017	y=3939.16-30.61x	0.9645	0.0179

**Figure 7:**
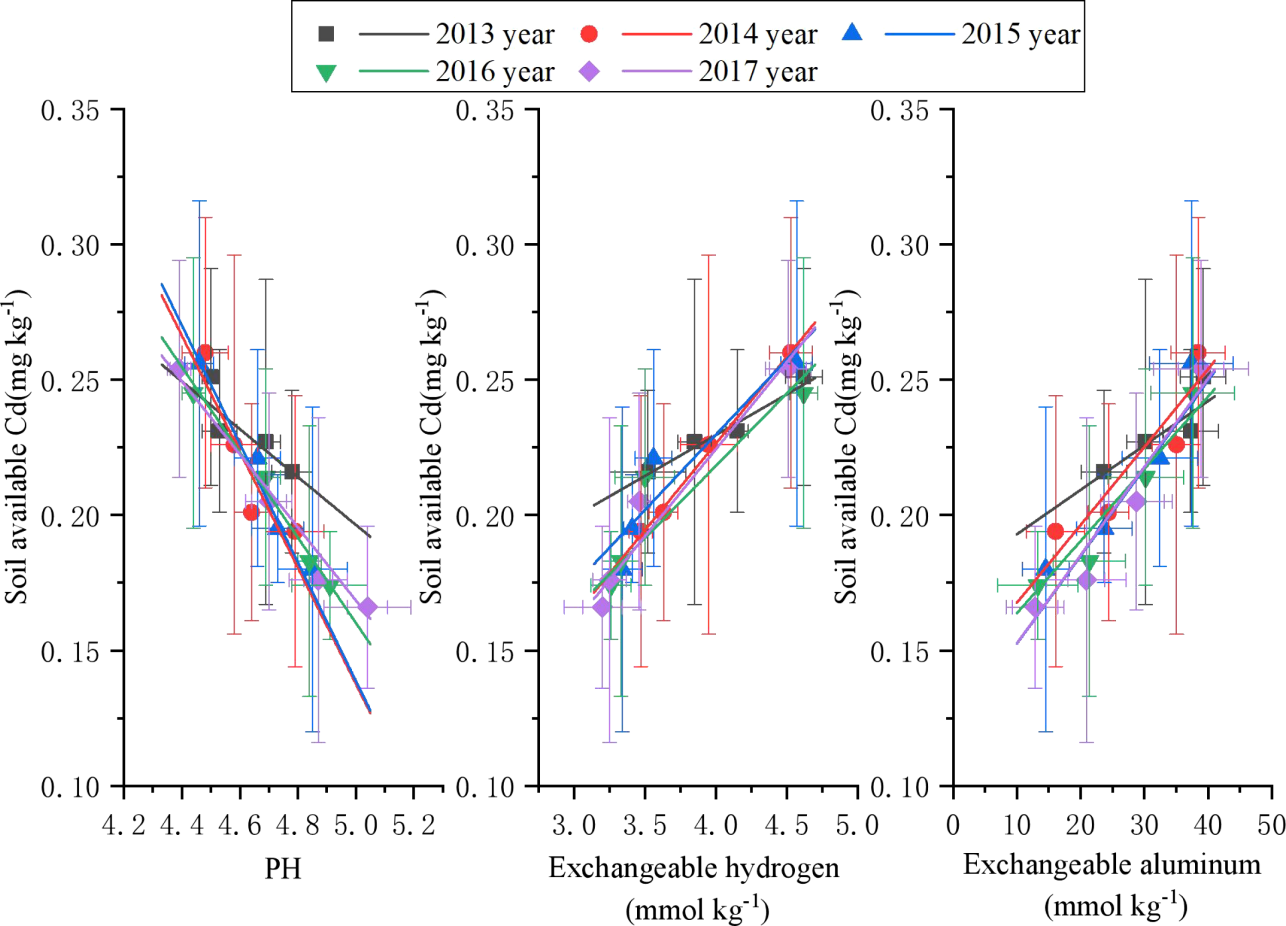
Correlation between soil available Cd and soil pH, exchangeable hydrogen, and exchangeable aluminum.

Soil pH, exchangeable hydrogen, and exchangeable aluminum exhibited opposite trends in relation to available Cd (**Figure 7**). Specifically, available Cd was negatively correlated with soil pH and positively correlated with exchangeable hydrogen and aluminum. In **Table 7**, for each 0.1 unit increase in pH, available Cd decreased by 0.06 to 0.12 mg kg^-^¹. Additionally, each 1 mmol kg^-^¹ increase in exchangeable hydrogen reduced available Cd by 0.004 to 0.11 mg kg^-^¹, while each 1 mmol kg^-^¹ increase in exchangeable aluminum decreased available Cd by 0.12 to 0.18 mg kg^-^¹.

**Table 7:** Fitted equations between soil available Cd and soil pH, exchangeable hydrogen, and exchangeable aluminum.

Indexes	Year	Equation	R^2^	P
pH	2013	y=-0.09 + 0.64x	0.74958	0.13422
	2014	y=-0.21 + 1.21x	0.80607	0.10219
	2015	y=-0.22 + 1.23x	0.93911	0.03092
	2016	y=-0.16 + 0.95x	0.98976	0.00513
	2017	y=-0.13 + 0.84x	0.98356	0.00825
Exchangeable hydrogen	2013	y=0.03 + 0.11x	0.96517	0.01757
	2014	y=0.03-0.03x	0.99609	0.00196
	2015	y=0.06 + 0.01x	0.82992	0.089
	2016	y=0.05 + 0.004x	0.82562	0.09136
	2017	y=0.06-0.03x	0.93655	0.03225
Exchangeable aluminum	2013	y=0.002 + 0.18x	0.77054	0.12219
	2014	y=0.003 + 0.14x	0.86271	0.07118
	2015	y=0.003 + 0.12x	0.90081	0.05089
	2016	y=0.003 + 0.14x	0.96109	0.01965
	2017	y=0.003 + 0.12x	0.95722	0.02162

## 4 Discussion

### 4.1 Effect of continuous oyster shell powder applications on crop yield

Peanut is an economically significant crop in China, providing a crucial source of both oil and protein. The annual peanut planting area in China exceeds 4.5 million hectares, accounting for approximately 35% of the total area sown for oil crops, with a production of over 17 million tons per year (Liu et al., 2020). In recent years, large-scale agricultural development efforts have improved and optimized peanut planting patterns, initiating a trend of intensive peanut cultivation. This continuous cropping has resulted in soil degradation and an overall decline in crop yields (Wang et al., 2013), attributed to slowed growth and development, impaired dry matter accumulation, reduced fruit setting rates, and decreased filled pod rates. Furthermore, this cultivation strategy can lower fertilizer utilization, impair nutrient absorption and utilization, and exacerbate pest and disease occurrences, thereby severely impacting both peanut yield and quality (Huang et al., 2013; Guo et al., 2024). Previous studies have shown that soil amendment applications can alleviate these obstacles, improving declining yields and mitigating poor growth conditions (Charles et al., 2022). In addition, it was shown that the contents of soil alkaline hydrolyzable nitrogen, available phosphorus, and available potassium did not change significantly under different rates of oyster shell powder, but there was no significant change (**Supplementary Table S1**, **Supplementary Table S2**, and **Supplementary Table S3**). This may have been due to the soil samples being collected after peanut harvesting. At this time, the application of oyster shell powder was more than 4 months away, and the absorption of nitrogen, phosphorus and potassium by the crops was added, the effect of the oyster shell powder on alkaline hydrolyzable nitrogen, available phosphorus, available potassium after peanut harvesting was negligible. Therefore, it was hypothesized that the contents of soil alkaline hydrolyzable nitrogen, available phosphorus, and available potassium could be increased following the application of oyster shell powder, as it is believed that oyster shell powder promotes the activation of soil nitrogen, phosphorus, and potassium.

### 4.2 Effect of continuous oyster shell powder applications on acidification control

Soil acidification in red soil drylands has become increasingly severe, likely due to continuous cropping and long-term chemical fertilizer applications. For instance, repetitive soybean cropping increases the release of organic acids, while nitrogen fixation results in greater absorption of cations than anions, leading to H^+^ secretion that acidifies the root zone soil, lowers nutrient utilization, and slows crop growth (Raven et al., 1990; Hinsinger et al., 2003). Moreover, the continuous application of chemical fertilizers, particularly nitrogen fertilizers, exacerbates soil acidification by promoting nitrate leaching and the depletion of base cations (Zhang et al., 2008; Cytryn et al., 2012).The experimental study showed that the pH of red soil dryland could be increased by 0.03-0.65 units by applying oyster shell powder, and the effect was more obvious with the increase of oyster shell powder dosage. The main reason is that oyster shell powder, which is deeply processed and mainly contains nutrients such as CO_3_^2−^ and exchangable calcium, is easy to neutralize and react with H+ in soil solution and produce CO_2_. By reducing the concentration of H^+^ and Al^3+^ in soil, soil acidity can be improved and acidification of red soil dryland can be controlled (Holland et al., 2018). Secondly, after the application of oyster shell powder, more cation exchange sites such as Ca^2+^ were provided, and Ca^2+^ occupied the original Al^3+^ position, Al^3+^ lost its charge, and Al^3+^ and H^+^ in the soil were reduced, thereby increasing the soil pH. This was consistent with the findings of Hati et al (Hati et al., 2008). The research results of Zhang et al (Zhang et al., 2018). showed that base ions adsorbed on colloids of acidic soil, such as NH_4_^+^, K^+^, Ca^2+^ and Mg^2+^, were mostly replaced by H^+^ and Al^3+^ into soil solution and leaching was lost. The application of oyster shell powder could increase the cation content that could be used for exchange, thus effectively repairing acidified soil.

### 4.3 Effect of Continuous Oyster Shell Powder Applications on Cadmium Immobilization

Heavy metals are common soil pollutants. Soil can also adsorb a certain amount of heavy metals (Yang et al., 2024). and the long-term use of pesticides containing these metals has been reported to elevate their concentrations in dryland soils beyond risk thresholds (Liang et al., 2017). The duration of dryland cultivation was significantly positively correlated with the accumulation of certain heavy metals (Jing et al., 2023). Since heavy metals are not easily degraded by soil microorganisms, their accumulation negatively affects crop quality and poses numerous health risks to humans, including both acute and chronic poisoning, as well as irreversible damage (Horiguchi et al., 2013; Nishijo et al., 2018). The experimental study showed that long-term application of soil oyster shell powder could reduce soil effective Cd by 7.96%-34.65%, and the effect became more obvious with the increase of the amount of soil oyster shell powder. In the treatment of heavy metal pollution, passivation restoration of soil by applying soil conditioner is a common method (Saengwilai et al., 2020; Li et al., 2023). The soil conditioner of oyster shell powder used in this study contains exchangeable calcium, which can improve soil pH, increase the negative charge carried by soil colloids, and increase the adsorption of heavy metal ions (Shaneen et al., 2013). As a result, the soil structure improves, the formation of aggregate structure accelerates, the availability of heavy metals in soil is reduced by improving the physical and chemical properties of the soil, and the water and fertilizer retention capacity of the soil is enhanced. Additionally, the growth environment of crop roots is improved, and the nutrient absorption capacity of roots increases (Shin et al., 2023). Furthermore, as peanut production gradually decreases, this may lead to a decrease in effective Cd content through conversion to non-absorbable Cd or absorption by peanuts. Therefore, future studies will focus on analyzing the Cd content in peanuts to better understand the long-term effects.

### 4.4 Relationship between acidification indicators, yield, and Cd with continuous oyster shell powder applications

Throughout our experiment, soil pH decreased annually due to continuous cropping. Oyster shell powder applications are known to effectively increase soil pH, enhancing the availability of nutrients such as phosphorus and potassium, promoting root development, and improving nutrient absorption (Fang et al., 2024). Our results demonstrated a negative correlation between soil exchangeable hydrogen, exchangeable aluminum, and pH, which was consistent with the findings of Huang et al (Huang et al., 2024). In upland red soil, peanut yield was positively correlated with pH but negatively correlated with soil exchangeable hydrogen and exchangeable aluminum. These results reflect the findings of Liao et al (Liao et al., 2018), which indicated that increasing pH promotes crop growth and yield in acidified soils. Conversely, Zeng et al (Zeng et al., 2017). reported that yield increases in acidified soils were dependent on the initial pH, with the greatest increase—up to 99%—occurring at a pH of 4.3 and decreasing as pH exceeds 5.8. Yield increases in acidified soils may also be related to crop variety.

After five consecutive years of oyster shell applications, soil available Cd showed a significant negative correlation with soil pH, exchangeable hydrogen, and exchangeable aluminum. This is consistent with the findings of Lin et al (Lin et al., 2023), which demonstrated that available soil Cd content decreased by 0.25%-18.8% when the pH of acidified soil increased by 1.17-1.69 units. Oyster shell powder applications play a key role in increasing soil pH, directly affecting available Cd content. This alteration significantly influences the speciation, mobility, and bioavailability of heavy metals in the soil. Acidic soils typically exhibit high solubility and bioavailability of Cd, as the metal is readily released from the solid phase into the soil solution under acidic conditions, elevating the risk of plant uptake and accumulation (Lian et al., 2022). The continuous application of oyster shell powder raises soil pH, reducing the concentration of available Cd by prompting its binding to soil particles and formation of insoluble compounds (Chen et al., 2021). Moreover, the rise in soil pH may also enhance the hydrolysis of other elements, such as iron, aluminum, and manganese, whose precipitation with Cd further reduces its bio-availability. Through the correct use of oyster shell powder, it can effectively improve the pH value of soil, reduce the harm of harmful elements such as Al^3+^ and Cd, improve acidic soil, and promote the healthy growth of crops. However, overapplication of oyster shell powder may lead to soil alkalization, which could affect crop growth. So, further research on the long-term effects of oyster shell application is essential, as potential improvements to soil quality could address the challenges of farmland degradation. In addition, it was known that soil microorganisms would be changed under long-term oyster shell powder applications, and key microbial communities may be involved in the migration and transformation of soil Cd. In the future, the changes of soil microbial communities in long-term application of oyster shell powder rates should be analyzed.

## 5 Conclusion

Our five-year field trial demonstrated that the application of oyster shell powder to red soil drylands significantly increased peanut yields and improved soil acidification. Compared to the control, applications of 750-2250 kg ha^-1^ of the amendment increased yields by 5.55%-37.01% and raised soil pH by 0.03-0.65 units. Continuous application of oyster shell powder showed that for every 0.1 unit increase in soil pH, peanut yields increased by 119.62-389.82 kg ha^-^¹, while available Cd content in the soil decreased by 0.06-0.12 mg kg^-^¹.

## Data Availability

The raw data supporting the conclusions of this article will be made available by the authors, without undue reservation.
