# Supplementary material for: Long-term oyster shell powder applications increase crop yields and control soil acidity and cadmium in red soil drylands
Source: Front Plant Sci. 2025 Feb 28;16:1506733. doi: 10.3389/fpls.2025.1506733 (PMC11906426; doi:10.3389/fpls.2025.1506733)
Supplement: Supplementary file 1 [file Table1.docx]

**Supplementary materials**

Table S1 Changes in soil alkaline hydrolyzable nitrogen with continuous oyster shell powder application

| Treatments | Soil alkaline hydrolyzable nitrogen (mg/kg) | | | | |
| --- | --- | --- | --- | --- | --- |
|  | 2013 | 2014 | 2015 | 2016 | 2017 |
| L0 | 46.58a | 44.28a | 40.44a | 38.43a | 39.45a |
| L750 | 50.05a | 50.82a | 46.59a | 52.15a | 51.22a |
| L1500 | 48.14a | 48.52a | 46.97a | 51.88a | 50.33a |
| L2250 | 47.79a | 49.31a | 47.37a | 48.16a | 49.54a |

Different lowercase letters indicate significant differences between treatments within the same year (p<0.05), The same to other Tables.

Table S2 Changes in soil available phosphorus with continuous oyster shell powder application

| Treatments | Soil available phosphorus (mg/kg) | | | | |
| --- | --- | --- | --- | --- | --- |
|  | 2013 | 2014 | 2015 | 2016 | 2017 |
| L0 | 19.25a | 18.10a | 17.60a | 18.32a | 19.22a |
| L750 | 17.75a | 21.65a | 21.05a | 23.48a | 20.33a |
| L1500 | 17.65a | 19.75a | 18.80a | 18.73a | 20.15a |
| L2250 | 21.55a | 20.15a | 23.95a | 21.88a | 21.34a |

Table S3 Changes in soil available potassium with continuous oyster shell powder application

| Treatments | Soil available potassium (mg/kg) | | | | |
| --- | --- | --- | --- | --- | --- |
|  | 2013 | 2014 | 2015 | 2016 | 2017 |
| L0 | 89.00a | 81.50a | 74.50a | 81.67a | 80.36a |
| L750 | 91.50a | 82.00a | 84.50a | 92.67a | 90.21a |
| L1500 | 91.50a | 81.00a | 81.50a | 88.00a | 89.34a |
| L2250 | 94.50a | 81.50a | 85.00a | 97.00a | 95.43a |
